# Causal Association of Dietary Habits With Thyroid Diseases: Univariate and Multivariate Mendelian Randomization Studies

**DOI:** 10.1002/fsn3.71473

**Published:** 2026-02-15

**Authors:** Ningwei Wang, Yunyi Yang, Jiawen You, Xiaoxiao Qu, Weijin Huang, Yanming He, Hongjie Yang

**Affiliations:** ^1^ Yueyang Hospital of Integrated Traditional Chinese and Western Medicine Shanghai University of Traditional Chinese Medicine Shanghai China

**Keywords:** dietary intakes, mendelian randomization, thyroid diseases

## Abstract

Dietary habits play a crucial role in everyday health, influencing both the onset and progression of diseases. Previous studies have shown some effects of dietary habits on certain thyroid diseases. However, comprehensive research on the causal relationship between dietary habits and thyroid diseases is lacking. The purpose of this study was to investigate the potential causal relationship between 45 genetically predicted dietary intake habits and thyroid diseases. We obtained GWAS data for 45 dietary intake habits from the UK Biobank and for 10 thyroid diseases from the FinnGen R12 database. We performed univariable Mendelian randomization (UVMR) using the inverse variance weighted (IVW) test as the primary test and corrected the results by false discovery rate (FDR) analysis. Additionally, multivariable Mendelian randomization analysis (MVMR) was further applied to assess independent effects of dietary habits. Linkage disequilibrium score regression (LDSC) was conducted on the FDR‐corrected significant findings. UVMR identified 28 potential causal associations, of which six remained significant after FDR correction. Cheese intake and alcohol consumption during meals were suggestively associated with a lower risk of hypothyroidism. Poultry consumption showed a potential association with a higher risk of nontoxic thyroid nodules, while moderate red wine intake appeared to be linked with a lower risk. In MVMR analyses adjusting for conventional confounders, the association between poultry consumption and both nontoxic nodules and goiter remained suggestive of an independent effect. These findings suggest potential causal links between dietary factors and thyroid diseases; however, considering the MR assumptions and horizontal pleiotropy, these estimates should be interpreted with caution, as they are hypothesis‐generating rather than prescriptive for clinical nutrition.

## Graphical abstract

In this study, we employ Univariate and Multivariate Mendelian Randomization to explore potential causal relationships between 45 dietary intake habits and 10 thyroid diseases. Our findings offer preliminary insights into the role of diet in thyroid disorder prevention and management.

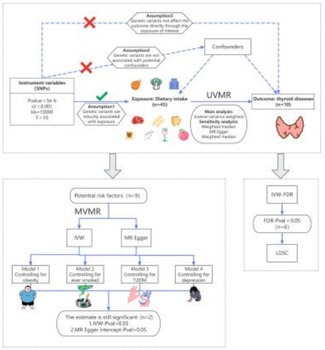

## 1 Introduction

Thyroid disorders are a major category of endocrine diseases that include both structural and functional abnormalities of the thyroid gland. These conditions encompass hypothyroidism (Chaker et al. 2017), hyperthyroidism (De Leo et al. 2016), Graves' disease (Davies et al. 2020), thyroiditis (Pearce et al. 2003), thyroid nodules (Grani et al. 2024), benign thyroid neoplasms and thyroid cancer (Chen et al. 2023). As the primary site of thyroxine (T4) and triiodothyronine (T3) production, the thyroid helps regulate functions in all bodily systems (Armstrong et al. 2025). A decade ago, the global population affected by thyroid disorders had already reached approximately 200 million (2012). Recent epidemiological data demonstrate a marked rise in the prevalence of thyroid diseases, with women showing a higher prevalence than men (Zhang, Wang, et al. 2023). Epidemiological studies report that hypothyroidism occurs in 0%–8% of populations (Zamwar and Muneshwar 2023), while hyperthyroidism demonstrates a prevalence between 0.2% and 1.3% (Taylor et al. 2018). Concurrently, rising incidences of thyroid nodules and thyroid cancer pose significant public health challenges (Huang et al. 2023; Uppal et al. 2023).

Accumulating evidence suggests associations between dietary factors and thyroid diseases. Dietary habits are an important part of daily life, and a balanced and nutritious diet plays a fundamental role in maintaining overall health and preventing diseases. Current studies report that a gluten‐free diet (GFD) may have beneficial effects on autoimmune thyroid diseases (Esfahani et al. 2024). Emerging evidence suggests that a Mediterranean‐style dietary pattern, primarily centered on plant‐based foods, may offer protective advantages against autoimmune thyroid disorders (Ruggeri et al. 2023). Higher consumption of raw vegetables has been linked to a lower likelihood of thyroid cancer (Jung et al. 2013). However, another study found that cruciferous vegetables, multivitamins, nitrites, pork, and poultry may increase thyroid cancer risk, whereas alcohol showed protective effects (Choi and Kim 2014). Taken together, research on the relationship between dietary patterns and thyroid diseases remains inconclusive.

Clarifying how diet influences thyroid diseases may help generate hypotheses for clinical prevention and treatment. Traditional randomized controlled trials (RCTs) often lack real‐world applicability because their rigid protocols do not accurately reflect clinical practice, which may limit the validity of their results. Moreover, the substantial resource demands and ethical constraints of RCTs frequently restrict their feasibility. To address these limitations, we employed Mendelian randomization (MR), an analytical approach that uses genetic variants as instrumental variables (IVs) to infer causal relationships (Palmer et al. 2012). By leveraging single‐nucleotide polymorphisms (SNPs) that are randomly assorted during meiosis, MR minimizes confounding and reverse causation biases (Davey Smith and Hemani 2014).

This study employed MR to examine the associations between 45 dietary factors and 10 thyroid diseases. We further conducted multivariable Mendelian randomization (MVMR) to assess independent contributions of specific exposure factors beyond conventional risk factors. In addition, linkage disequilibrium score regression (LDSC) was applied to evaluate genetic correlations. These findings are intended to provide preliminary hypotheses for further investigation into the potential links between diet and thyroid diseases.

## 2 Methods

### 2.1 Study Design

**FIGURE 1:**
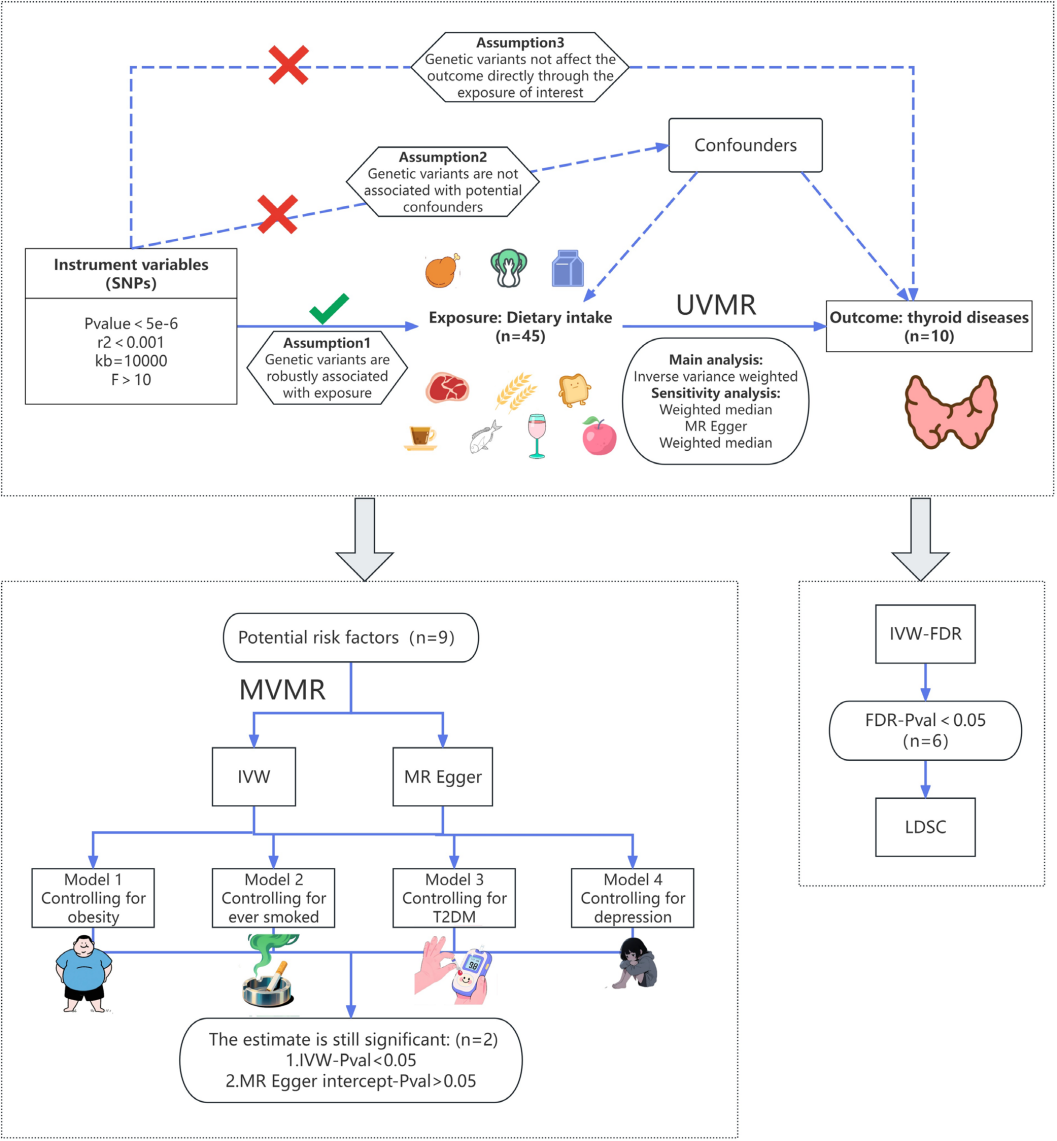
Schematic of the study design.

Figure 1 illustrates the study workflow. We used univariable Mendelian randomization (UVMR) analyses to examine the potential associations between 45 dietary habits and 10 thyroid diseases. The validity of MR rests on three core assumptions: IVs must be strongly associated with exposures, independent of outcomes, and unrelated to confounders (Boef et al. 2015). To evaluate whether associations identified in the UVMR could be explained by conventional risk factors for thyroid diseases, we performed MVMR models. All analyses were conducted in R version 4.3.3, using the “TwoSampleMR”, “MendelianRandomization” and “MR‐PRESSO” packages.

### 2.2 Data Sources

Dietary habit data are summarized in Table S1. We obtained these data from the UK Biobank via the IEU OpenGWAS project, which contains lifestyle and physical health information from approximately 500,000 participants (Bycroft et al. 2018). Dietary intake, including consumption of cereals, bread, fruit and vegetables, meat and fish, dairy products, beverages, salt, and other dietary items, were collected using a touchscreen questionnaire. The GWAS summary statistics for 10 thyroid conditions were obtained from the FinnGen R12 release, including hypothyroidism (strict autoimmune), hypothyroidism (drug reimbursement), autoimmune hyperthyroidism, Graves' disease (strict definition), thyroiditis, nontoxic single thyroid nodule, nontoxic goiter/thyroid nodule, benign neoplasm of thyroid gland, papillary adenocarcinoma of thyroid gland (excluding all cancers), and malignant neoplasm of thyroid gland (excluding all cancers) (Kurki et al. 2023). The diagnostic criteria and additional information for 10 thyroid diseases are available at https://risteys.finregistry.fi/. In addition, we incorporated conventional risk factors for thyroid diseases, including obesity (Jiang et al. 2021), ever smoked (Howe et al. 2022), type 2 diabetes mellitus (T2DM) (Loya et al. 2025), and major depression (Howard et al. 2019). The corresponding summary data can be found at https://www.ebi.ac.uk/gwas/ and https://gwas.mrcieu.ac.uk/, respectively. Further details are provided in Table 1.

**TABLE 1:** Description of GWAS data sources for each phenotype.

Dataset type	Variable	ID or GWAS ID	Samplesize	Journal	Population	Sex
Exposure	Dietary habits	See Table S1		Nature	European	Males and females
	Obesity	GCST90043672	456,348	Nature	European	Males and females
	Ever smoke	ieu‐b‐4858	99,996	Nature	European	Males and females
	T2DM	GCST90468151	394,626	Nature	European	Males and females
	Major depression	ieu‐b‐102	500,199	Nature	European	Males and females
Outcome	Hypothyroidism, strict autoimmune	finngen_R12_E4_HYTHY_AI_STRICT	417,391	Nature	European	Males and females
	Hypothyroidism, drug reimbursement	finngen_R12_HYPOTHY_REIMB	128,104	Nature	European	Males and females
	Autoimmune hyperthyroidism	finngen_R12_AUTOIMMUNE_HYPERTHYROIDISM	373,106	Nature	European	Males and females
	Graves disease, strict definition	finngen_R12_E4_GRAVES_STRICT	500,348	Nature	European	Males and females
	Thyroiditis	finngen_R12_THYROIDITIS	489,451	Nature	European	Males and females
	Nontoxic single thyroid nodule	finngen_R12_E4_GOITRENOD	426,983	Nature	European	Males and females
	Nontoxic goiter/thyroid nodule	finngen_R12_E4_NONTOXIC_THYROID	500,348	Nature	European	Males and females
	Benign neoplasm of thyroid gland	finngen_R12_CD2_BENIGN_THYROID	500,348	Nature	European	Males and females
	Malignant neoplasm of thyroid gland	finngen_R12_C3_THYROID_GLAND_EXALLC	381,608	Nature	European	Males and females
	Papillary adenocarcinoma of thyroid gland	finngen_R12_C3_THYROID_PAPILLARY_ADENO_EXALLC	373,106	Nature	European	Males and females

### 2.3 IVs Selection

Given the limited number of SNPs associated with dietary exposures at the conventional genome‐wide significance threshold (*p* < 5 × 10^−8^), a relaxed threshold (*p* < 5 × 10^−6^) was applied to retain an adequate number of variants as potential IVs. SNPs in linkage disequilibrium (LD; *R* ^2^ > 0.001 within 10,000 kb) were pruned to maintain relative independence among SNPs (Hemani et al. 2018). SNPs with *F* < 10 were regarded as weak IVs and were excluded from subsequent analyses to minimize instrumental bias (Burgess et al. 2013). To reduce the influence of confounding bias and possible reverse causation, SNPs showing strong associations with the outcomes (*p* < 5 × 10^−5^) were also excluded. Harmonization was performed to remove palindromic sequences and mismatched SNPs, ensuring adherence to the assumptions of Independence and Exclusivity (Pierce et al. 2011). Tables S25–S34 provided the complete dataset of selected SNPs.

### 2.4 UVMR Analysis

We used the inverse variance weighted (IVW) method as the primary analytical strategy to estimate associations, providing a robust assessment of the investigated relationships and enabling more reliable conclusions (Burgess et al. 2013, 2015). Heterogeneity was assessed using Cochran's *Q*‐test: fixed‐effects IVW (IVW‐FE) was applied when *P*_Q_ > 0.05, and random‐effects IVW (IVW‐MRE) when *P*_Q_ < 0.05 (Burgess et al. 2013). To evaluate robustness, we performed sensitivity analyses using MR‐Egger regression, the weighted median, and MR‐PRESSO, and included estimates whose directions were concordant with the IVW results. MR‐Egger was used to identify and adjust for potential horizontal pleiotropy, with its slope providing a relatively robust estimate in MR (Burgess and Thompson 2017). The weighted median approach can tolerate up to 50% of invalid IVs and provides more reliable causal estimates under certain conditions (Bowden et al. 2016; Hartwig et al. 2017). MR‐PRESSO identified outliers and provided corrected estimates to reduce distortion from horizontal pleiotropy (Verbanck et al. 2018). Moreover, we applied false discovery rate (FDR) correction to IVW *p*‐values, defining *p*_FDR_ < 0.05 as statistically robust and *p*_FDR_ > 0.05 as suggestive (Benjamini and Hochberg 1995).

### 2.5 MVMR Analysis

UVMR assesses the overall effect of an exposure on an outcome, whereas MVMR estimates the independent effect of each exposure after accounting for other exposures (Burgess and Thompson 2015; Sanderson et al. 2019). For MVMR, IVW remained our primary analytical approach. We incorporated established correlates of thyroid diseases, including obesity, ever smoked, T2DM, and major depression (Kalra et al. 2019; Tang et al. 2017; Walczak and Sieminska 2021; Zamora et al. 2025; Zhang et al. 2024), to assess whether the observed associations between dietary habits and thyroid disease were influenced by these confounders. The IVs selected and parameter settings for MVMR were consistent with those used in UVMR.

### 2.6 Genetic Correlation Analysis

Finally, we analyzed the results that remained significant after FDR correction using the linkage disequilibrium score regression (LDSC), a method commonly applied to estimate the genetic correlation (Rg) between complex traits and diseases (Bulik‐Sullivan, Finucane, et al. 2015; Bulik‐Sullivan, Loh, et al. 2015). A *p*‐value < 0.05 was considered evidence of a genetic correlation.

## 3 Results

### 3.1 Results of UVMR Analysis

**FIGURE 2:**
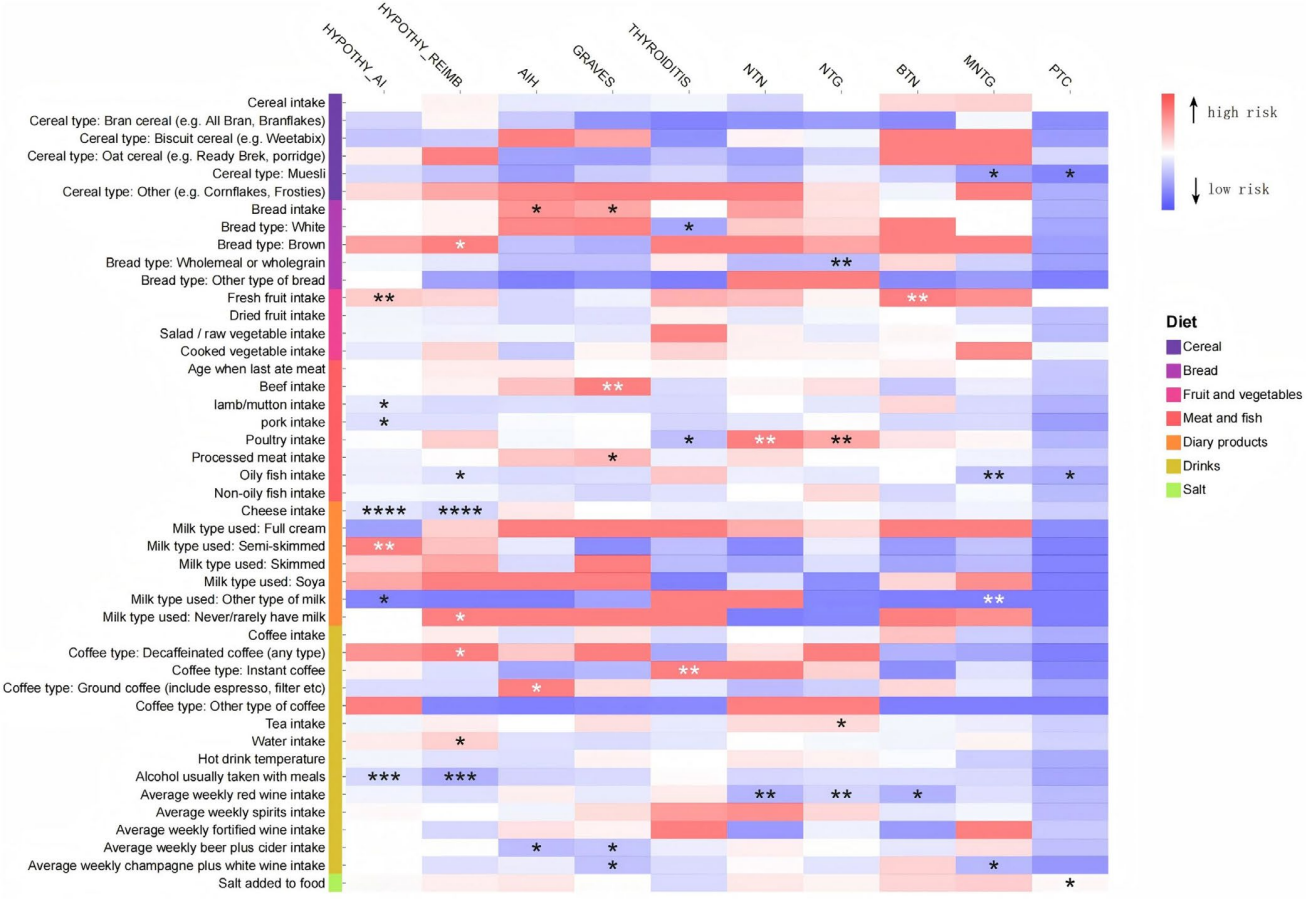
This heat map illustrates the causal associations between 45 dietary intake habits and 10 thyroid disorders, as determined by the IVW method. More asterisks indicate greater significance.

**FIGURE 3:**
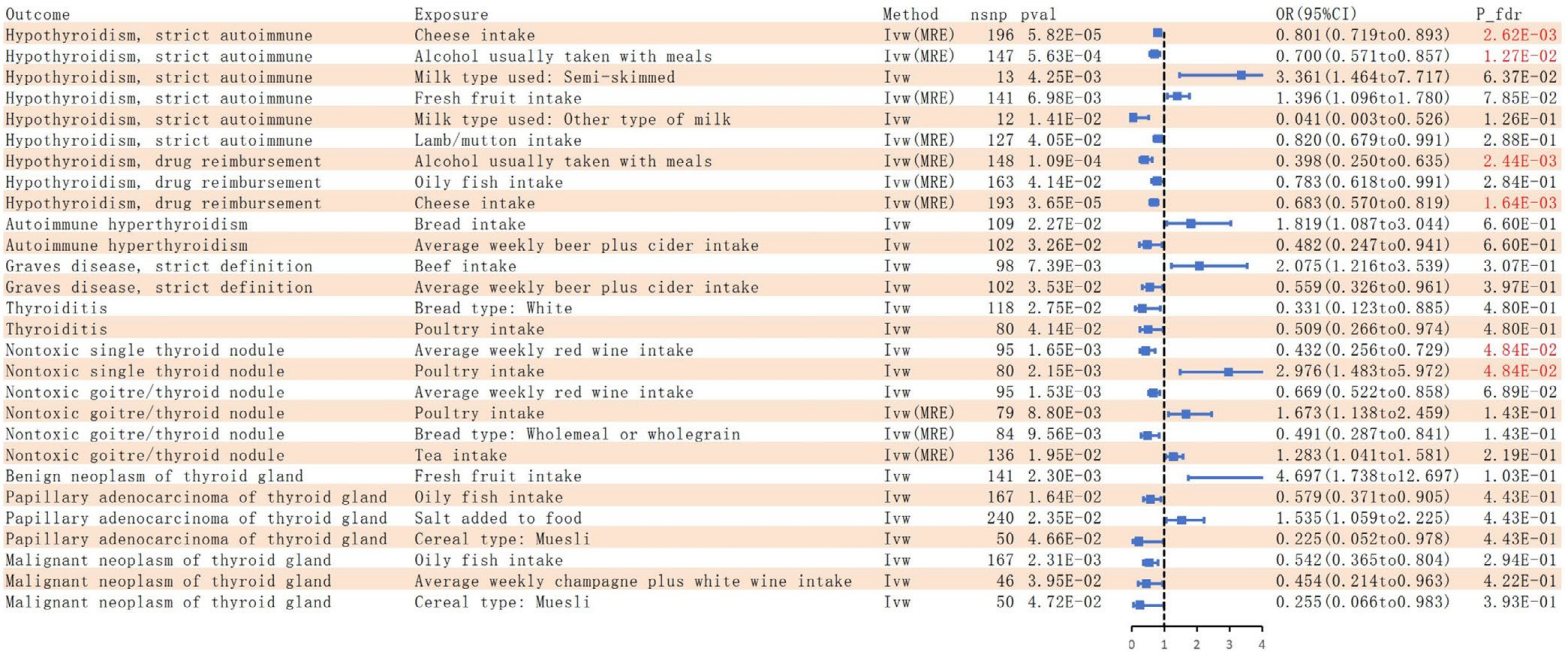
Forest plot of the causality between dietary intake habits and thyroid diseases using IVW method. CI, confidence interval; FDR, false discovery rate; IVW, Inverse variance weighted; MRE, Multiplicative random effects; OR, odds ratio.

We conducted UVMR to examine the potential relationship between 10 types of thyroid diseases and 45 common dietary intake habits. Furthermore, all *p*‐values were corrected using FDR, as shown in Figure 2 and Tables S2–S11 and S22. To verify the robustness of the findings, we employed sensitivity analyses (see Tables S12–S21). The IVW results identified 28 dietary habits showing potential associations with thyroid diseases, of which 6 remained statistically significant after FDR adjustment (*p*_FDR_ < 0.05). For hypothyroidism (strict autoimmune), four dietary exposures showed inverse associations, and cheese intake (OR = 0.801, 95% CI: 0.719–0.893, *p* < 0.001, *p*_FDR_ = 0.003) together with alcohol usually taken with meals (OR = 0.700, 95% CI: 0.571–0.857, *p* < 0.001, *p*_FDR_ = 0.013) remained significant after FDR correction. Signals that did not survive FDR correction included milk type used: other type of milk (OR = 0.041, 95% CI: 0.003–0.526, *p* = 0.014) and lamb/mutton intake (OR = 0.820, 95% CI: 0.679–0.991, *p* = 0.041). Positive associations were observed for milk type: semi‐skimmed (OR = 3.361, 95% CI: 1.464–7.717, *p* = 0.004) and fresh fruit intake (OR = 1.396, 95% CI: 1.096–1.780, *p* = 0.007). For hypothyroidism (drug reimbursement), alcohol usually taken with meals (OR = 0.398, 95% CI: 0.250–0.635, *p* < 0.001, *p*_FDR_ = 0.002) and cheese intake (OR = 0.683, 95% CI: 0.570–0.819, *p* < 0.001, *p*_FDR_ = 0.002) showed robust inverse associations, and oily fish intake was also inversely associated (OR = 0.783, 95% CI: 0.618–0.991, *p* = 0.041). Bread intake (OR = 1.819, 95% CI: 1.087–3.044, *p* = 0.023) was positively associated with autoimmune hyperthyroidism, whereas average weekly beer plus cider consumption (OR = 0.482, 95% CI: 0.247–0.941, *p* = 0.033) showed an inverse association. Beef intake (OR = 2.075, 95% CI: 1.216–3.539, *p* = 0.007) was positively associated with Graves' disease, while average weekly beer plus cider intake (OR = 0.559, 95% CI: 0.326–0.961, *p* = 0.035) was inversely associated. For thyroiditis, both white bread (OR = 0.331, 95% CI: 0.123–0.885, *p* = 0.028) and poultry intake (OR = 0.509, 95% CI: 0.266–0.974, *p* = 0.041) showed inverse associations. Poultry intake (OR = 2.976, 95% CI: 1.483–5.972, *p* = 0.002, *p*_FDR_ = 0.048) was positively associated with nontoxic single thyroid nodule, while average weekly red wine intake (OR = 0.432, 95% CI: 0.256–0.729, *p* = 0.002, *p*_FDR_ = 0.048) was inversely associated; both remained significant after FDR adjustment. For nontoxic goiter and thyroid nodule, poultry (OR = 1.673, 95% CI: 1.138–2.459, *p* = 0.009) and tea intake (OR = 1.283, 95% CI: 1.041–1.581, *p* = 0.020) showed positive associations, whereas average weekly red wine intake (OR = 0.669, 95% CI: 0.522–0.858, *p* = 0.002) and wholemeal or wholegrain (OR = 0.491, 95% CI: 0.287–0.841, *p* = 0.010) showed inverse associations. Fresh fruit intake (OR = 4.697, 95% CI: 1.738–12.697, *p* = 0.002) was positively associated with benign neoplasm of thyroid gland. For papillary adenocarcinoma of the thyroid gland, oily fish intake (OR = 0.579, 95% CI: 0.371–0.905, *p* = 0.016) and muesli (OR = 0.225, 95% CI: 0.052–0.978, *p* = 0.047) were inversely associated, while salt added to food (OR = 1.535, 95% CI: 1.059–2.225, *p* = 0.024) was positively associated. For malignant neoplasm of thyroid gland, inverse associations were observed for oily fish (OR = 0.542, 95% CI: 0.365–0.804, *p* = 0.002), average weekly champagne plus white wine intake (OR = 0.454, 95% CI: 0.214–0.963, *p* = 0.040) and muesli (OR = 0.255, 95% CI: 0.066–0.983, *p* = 0.047). Figure 3 summarizes the IVW results after sensitivity analyses. The MR‐Egger intercepts indicated no evidence of horizontal pleiotropy, and the MR‐PRESSO results remained statistically significant, suggesting that the observed associations were generally robust, although residual bias cannot be entirely ruled out.

### 3.2 Results of MVMR Analysis

**FIGURE 4:**
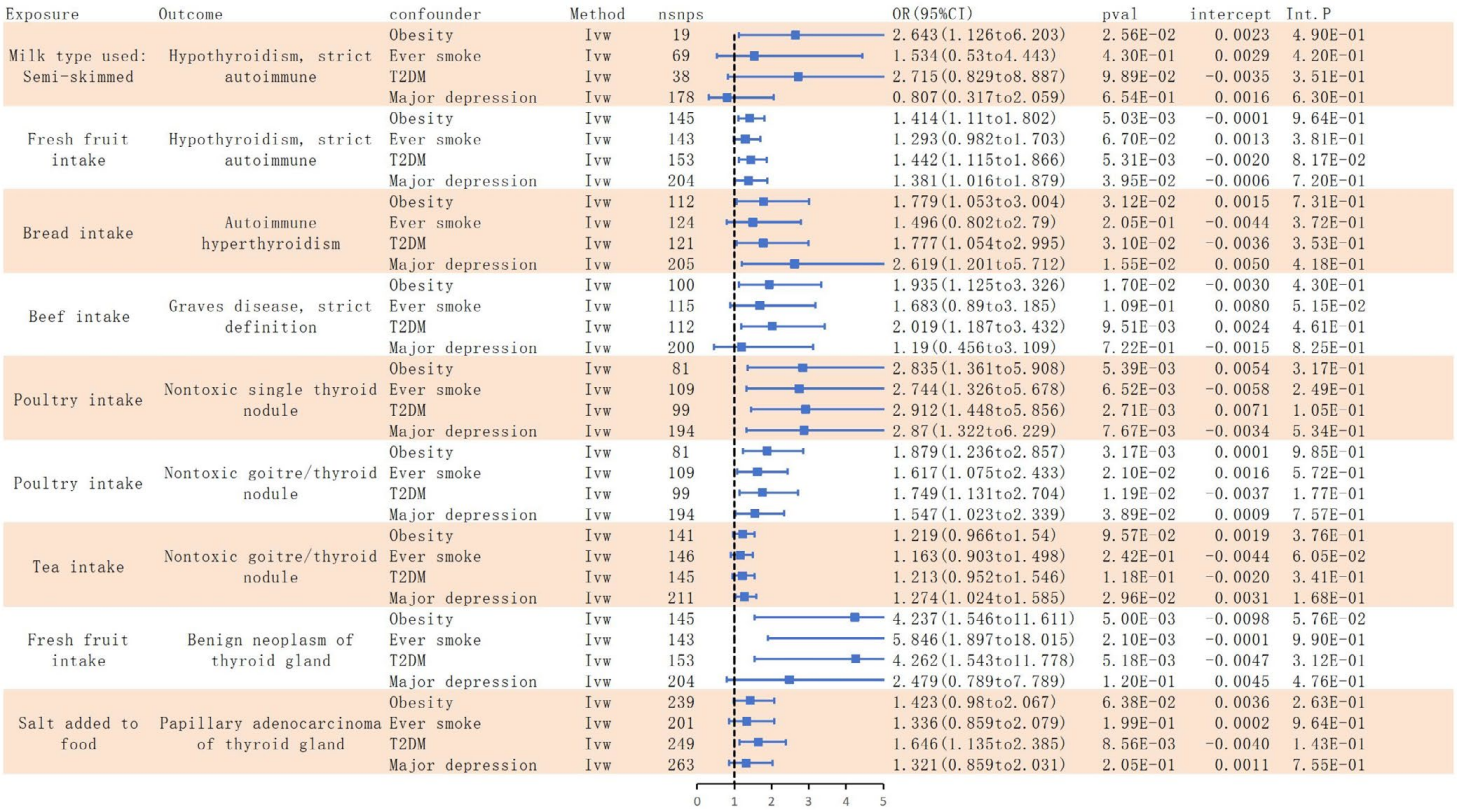
Forest plot of multivariate analysis for dietary risk factors, adjusted for traditional risk factors.

The MVMR results are shown in Figure 4. The UVMR results identified nine dietary habits that were associated with higher odds of thyroid diseases. The MVMR analysis further adjusted for four common correlates of thyroid disease, namely obesity, smoking history, T2DM, and depression. Comprehensive data are provided in Table S23. After adjustment, two associations remained statistically significant, involving poultry intake with nontoxic single thyroid nodule and with nontoxic goiter/thyroid nodule. The remaining seven associations were attenuated to non‐significance, suggesting that obesity, smoking, T2DM, and depression may account for part of the UVMR findings. Moreover, the MR‐Egger intercept tests did not reach statistical significance, providing no evidence of horizontal pleiotropy.

### 3.3 Results of Rg Analysis

To explore the genetic correlations between dietary habits and thyroid disorders, we conducted LDSC analyses on associations that remained significant after FDR correction. LDSC analyses revealed that alcohol usually taken with meals (Rg = −0.069, *p* = 0.024) and cheese intake (Rg = −0.072, *p* = 0.020) showed significant negative genetic correlations with hypothyroidism (strict autoimmune). In addition, alcohol usually taken with meals (Rg = −0.009, *p* = 0.011) and cheese intake (Rg = −0.176, *p* < 0.001) also exhibited negative genetic correlations with hypothyroidism (drug reimbursement). In contrast, average weekly red wine intake and poultry intake were not significantly genetically correlated with nontoxic single thyroid nodule, suggesting that the observed associations may not be attributable to underlying genetic factors. Detailed estimates are provided in Tables S24 and 2.

**TABLE 2:** Results of LDSC for all positive results after FDR correction in UVMR.

Phenotype 1	Phenotype 2	Rg	SE	*p*
Alcohol usually taken with meals	Hypothyroidism, strict autoimmune	−6.95E‐02	3.08E‐02	2.42E‐02
Cheese intake	Hypothyroidism, strict autoimmune	−7.27E‐02	3.13E‐02	2.02E‐02
Alcohol usually taken with meals	Hypothyroidism, drug reimbursement	−9.21E‐02	3.60E‐02	1.06E‐02
Cheese intake	Hypothyroidism, drug reimbursement	−1.76E‐01	3.34E‐02	1.41E‐07
Average weekly red wine intake	Nontoxic single thyroid nodule	2.52E‐01	1.60E‐01	1.15E‐01
Poultry intake	Nontoxic single thyroid nodule	3.22E‐01	1.85E‐01	8.12E‐02

Abbreviations: LDSC, linkage disequilibrium score regression; Rg, genetic correlation; SE, standard error.

## 4 Discussion

We conducted a systematic and comprehensive MR analysis to investigate the associations between 45 dietary habits and 10 thyroid disorders. To our knowledge, there have been no MR analyses related to this. Our research filled the gap in this aspect. We identified 28 suggestive associations, six of which remained statistically significant after FDR correction. The key findings were as follows: Cheese intake and alcohol consumption during meals were suggestively associated with a lower risk of hypothyroidism. Poultry consumption showed a potential association with a higher risk of nontoxic thyroid nodules, while moderate red wine intake appeared to be linked with a lower risk. In MVMR analyses adjusting for conventional confounders, the association between poultry consumption and both nontoxic nodules and goiter remained suggestive of an independent effect. Furthermore, the LDSC results further revealed that cheese intake and alcohol consumed during meals showed significant genetic correlations with hypothyroidism. We also identified several other dietary factors potentially associated with thyroid disorders, including oily fish, muesli, fresh fruit, bread, salt, tea, and other foods, which are discussed in detail in the following sections.

In this study, genetically proxied higher alcohol consumption was associated with lower odds of several thyroid disorders, including hypothyroidism, autoimmune hyperthyroidism, Graves' disease, thyroid cancer, nontoxic thyroid nodule, and goiter. In agreement with our results, a previous cross‐sectional study conducted in Greece documented that the incidence of thyroid disorder declined as alcohol consumption increased in both male and female populations (Voulgari et al. 2022). Evidence from case–control and prospective studies further suggests that moderate alcohol intake may have a protective effect against autoimmune thyroid diseases (Carlé et al. 2012; Effraimidis et al. 2012; Wiersinga 2016). A significant inverse association and genetic correlation were observed between alcohol usually taken with meals and hypothyroidism. A Korean cohort study found that appropriate alcohol intake is negatively associated with hypothyroidism (Kim and Park 2023). Another experimental study has demonstrated that hypothyroidism increases voluntary ethanol consumption and alters the expression of ethanol‐metabolizing enzymes such as ADH1 and ALDH2 (Echeverry‐Alzate et al. 2019). In our genetic analyses, average weekly beer plus cider intake was inversely associated with Graves' disease and autoimmune hyperthyroidism, in line with findings from a Danish case–control study showing reduced risk of Graves' hyperthyroidism among moderate drinkers, independent of age and sex (Carlé et al. 2013). Possible biological mechanisms underlying these associations may involve alcohol‐induced inhibition of natural killer cell activity (Bounds et al. 1994; Charpentier et al. 1984), reduction in antibody production (Aldo‐Benson 1989), and increased generation of gut‐derived anti‐inflammatory fatty acids, such as, short‐chain and polyunsaturated fatty acids (Caslin et al. 2021). We also observed negative associations of genetically predicted red‐wine intake with nontoxic thyroid nodule and goiter. Previous Chinese studies, including one cross‐sectional study and the baseline survey of a cohort study, reported that alcohol consumption is a protective factor against thyroid nodules (Li et al. 2024; Wan et al. 2023). Balhara et al.'s systematic evaluation arrived at comparable conclusions. Experimental data suggest that alcohol can exert a direct toxic impact on thyroid cells, potentially suppressing thyroid activity and reducing gland volume, which explains the historical use of ethanol ablation in treating thyroid nodules (Balhara and Deb 2013). Moreover, resveratrol, a polyphenol found in red wine, may be beneficial to human health due to its anti‐inflammatory, antioxidant, and antiproliferative effects. Although current evidence does not establish a direct relationship between resveratrol and thyroid nodules, several experimental studies have suggested that resveratrol may suppress thyroid cancer cell proliferation and tumor progression (Du and Shen 2023; Lu et al. 2022; Unal Kocabas et al. 2024). As this study suggests, genetically proxied consumption of champagne plus white wine showed an inverse association with malignant neoplasm of the thyroid gland. Although genetically predicted alcohol consumption showed inverse associations with certain thyroid diseases, these results should not be interpreted as evidence supporting alcohol use for prevention. Given its well‐known health risks, any apparent protective associations should be considered exploratory and require confirmation in mechanistic and prospective studies.

Cheese is a fermented dairy product rich in high‐quality proteins (mainly casein), lipids, essential minerals, and vitamins, as well as probiotics and bioactive molecules, which may confer health benefits (Zhang, Dong, et al. 2023). In our analyses, genetically proxied higher cheese intake was associated with lower odds of hypothyroidism, and this association was further supported by LDSC. From a biological perspective, iodine is essential for thyroid hormone synthesis, with major dietary sources including dairy products, seafood, and iodized salt. Inadequate iodine intake may lead to hypothyroidism (Niwattisaiwong et al. 2017). A cross‐sectional study in pregnant women from Saudi Arabia suggested that higher dairy consumption might be linked to a lower risk of hypothyroidism during pregnancy (Refaat and Azzeh 2021). Similarly, another pediatric cross‐sectional study indicated that higher dairy or cheese intake was associated with a reduced risk of Hashimoto's thyroiditis, a major cause of hypothyroidism (Özgüç Çömlek and Körez 2025). These findings are, however, observational or context‐specific and should be interpreted and generalized with caution. Cheese as a fermented dairy food, which may help maintain gut microbiota balance (Żółkiewicz et al. 2020) and possesses potential anti‐inflammatory, antioxidant, and immunomodulatory effects (Hess et al. 2021; Rangel et al. 2023).

Our MR analyses indicated that genetically proxied higher poultry intake was associated with an increased likelihood of nontoxic thyroid nodules and goiter. These associations persisted in MVMR after adjustment for obesity, T2DM, smoking, and depression, suggesting that the effects may be independent of these correlated factors. Although no prior studies have directly examined this relationship, a cross‐sectional study conducted in the U.S. reported that poultry intake was associated with higher urinary total arsenic levels, likely due to the historical use of arsenic‐based feed additives such as roxarsone and nitarsone. Inorganic arsenic is a well‐recognized carcinogen (Nigra et al. 2017). A case–control study from Kuwait reported that frequent chicken consumption was independently and positively correlated with thyroid cancer, showing a clear dose–response relationship (Memon et al. 2002). Cooking practices may also contribute: cured, smoked, or high‐temperature meat preparation can generate polycyclic aromatic hydrocarbons (PAHs) and other harmful compounds (Turesky 2018). Conversely, our analyses suggested an inverse association between poultry intake and the occurrence of thyroiditis. Consistent with this, observational studies have reported a lower incidence of autoimmune thyroiditis among individuals with higher poultry consumption compared with those consuming red or processed meats (Ruggeri et al. 2021).

In the present study, higher oily‐fish intake was inversely associated with hypothyroidism, papillary thyroid adenocarcinoma, and malignant thyroid neoplasms. From a biological perspective, oily fish are rich in omega‐3 polyunsaturated fatty acids, which may help modulate inflammatory pathways (Calder 2017). Pooled case–control evidence suggests that higher fish intake does not increase thyroid cancer risk and may be beneficial in iodine‐deficient areas (Bosetti et al. 2001). Prospective cohort studies have shown that higher consumption of oily fish is associated with lower risks of postpartum autoimmune thyroid disease and thyroiditis, both of which may lead to hypothyroidism (Benvenga et al. 2016, 2019). Fish also contributes iodine, essential for thyroxine synthesis (Abuduwaili et al. 2024). We also observed a positive association between adding salt to food and papillary thyroid carcinoma. After adjustment for smoking, obesity, and depression, the association weakened, suggesting possible mediation through these correlated factors. A prior large retrospective study reported that daily salt intake exceeding 5 g was associated with higher thyroid cancer risk (Wang et al. 2021). We also noted suggestive associations between fresh fruit, milk type, tea, bread, beef, and lamb intake and certain thyroid disorders. Because no prior thyroid‐specific literature has addressed these exposures, these findings should be considered exploratory and require validation in future studies.

Taken together, while several observational and experimental studies support our findings, the overall evidence remains limited by heterogeneity and modest statistical power. Among the cited studies, most were cross‐sectional or cohort in design, with limited RCTs or meta‐analyses to support causal inference, and their results might be influenced by sample size, geographic variation, risk of bias, or unmeasured confounding. Therefore, these associations should be viewed as preliminary within the broader hierarchy of evidence.

Several limitations should be acknowledged. First, dietary exposures were derived from GWAS based on self‐reported questionnaires, which are inherently prone to measurement error. Second, the study population consisted mainly of individuals of European ancestry, limiting generalizability to other populations and precluding subgroup analyses by age and sex. Third, while MR‐PRESSO analysis ruled out detectable horizontal pleiotropy, residual pleiotropy and unmeasured confounding cannot be excluded. Moreover, the sources of heterogeneity observed in some outcomes were not fully explored. Importantly, MR reflects associations based on lifelong genetic predispositions rather than short‐term dietary behaviors. Therefore, the current results provide evidence suggestive of potential causal relationships but should be regarded as exploratory and hypothesis‐generating. These findings should not be interpreted as direct guidance for nutritional interventions. Further validation through prospective and experimental studies in diverse populations is essential before drawing clinical or dietary conclusions.

## 5 Conclusion

This MR study explored potential causal relationships between genetically predicted dietary habits and thyroid disorders using large‐scale genetic data. We identified 28 associations of interest in UVMR, six of which remained statistically significant after FDR correction. MVMR suggested that poultry intake might be independently associated with an increased risk of nontoxic thyroid nodules and goiter, while LDSC revealed genetic correlations between cheese intake and alcohol consumption with hypothyroidism. These findings should be interpreted cautiously as exploratory and hypothesis‐generating rather than definitive evidence of causality. Further validation of these observed associations through prospective or interventional studies, as well as replication in independent and ethnically diverse populations, is warranted. Future research should also investigate the biological mechanisms underlying these relationships and explore broader dietary patterns rather than single food components. If validated, these findings may eventually inform evidence‐based nutritional guidance for thyroid disease prevention and management.

## Author Contributions

**Ningwei Wang:** conceptualization (equal), data curation (equal), methodology (equal), software (equal), visualization (equal), writing – original draft (lead). **Yunyi Yang:** data curation (equal), resources (equal). **Jiawen You:** validation (equal), visualization (equal). **Xiaoxiao Qu:** methodology (equal), visualization (equal). **Weijin Huang:** methodology (equal), software (equal), validation (equal). **Yanming He:** supervision (equal). **Hongjie Yang:** writing – review and editing (lead).

## Funding

This work was supported by the Shanghai Shenkang Three‐Year Action Plan for Promoting Clinical Skills and Clinical Innovation in Municipal Hospitals‐Research Physician Training in Innovative and Translational Capabilities Project (SHDC2022CRD009).

## Ethics Statement

Ethical review and approval were not required for this study because the data used in this study were public, anonymized, and de‐identified.

## Consent

The authors have nothing to report.

## Conflicts of Interest

The authors declare no conflicts of interest.

## Supporting information


**Table S1:** fsn371473‐sup‐0001‐TableS1‐S34.xlsx.

## Data Availability

The data that support the findings of this study are openly available at https://risteys.finregistry.fi/, https://www.ebi.ac.uk/gwas/ and https://gwas.mrcieu.ac.uk/.

## References

[fsn371473-bib-0001] Abuduwaili, G. , J. Huang , Y. Ma , and H. Sun . 2024. “Adult Dietary Patterns and Their Association With Iodine Nutrition Levels and Thyroid Function: A Cross‐Sectional Study.” Public Health Nutrition 28, no. 1: e4. 10.1017/s1368980024002404. 39600224 PMC11736648

[fsn371473-bib-0002] Aldo‐Benson, M. 1989. “Mechanisms of Alcohol‐Induced Suppression of B‐Cell Response.” Alcoholism, Clinical and Experimental Research 13, no. 4: 469–475. 10.1111/j.1530-0277.1989.tb00358.x. 2529792

[fsn371473-bib-0003] Armstrong, M. , E. Asuka , and A. Fingeret . 2025. “Physiology, Thyroid Function.” In StatPearls. StatPearls Publishing. 30725724

[fsn371473-bib-0004] Balhara, Y. P. , and K. S. Deb . 2013. “Impact of Alcohol Use on Thyroid Function.” Indian Journal of Endocrinology and Metabolism 17, no. 4: 580–587. 10.4103/2230-8210.113724. 23961472 PMC3743356

[fsn371473-bib-0005] Benjamini, Y. , and Y. Hochberg . 1995. “Controlling the False Discovery Rate: A Practical and Powerful Approach to Multiple Testing.” Journal of the Royal Statistical Society. Series B, Statistical Methodology 57, no. 1: 289–300.

[fsn371473-bib-0006] Benvenga, S. , M. T. Vigo , D. Metro , R. Granese , R. Vita , and M. Le Donne . 2016. “Type of Fish Consumed and Thyroid Autoimmunity in Pregnancy and Postpartum.” Endocrine 52, no. 1: 120–129. 10.1007/s12020-015-0698-3. 26306774

[fsn371473-bib-0007] Benvenga, S. , R. Vita , F. Di Bari , R. Granese , D. Metro , and M. Le Donne . 2019. “Stable Consumption of Swordfish Favors, Whereas Stable Consumption of Oily Fish Protects From, Development of Postpartum Thyroiditis.” Endocrine 65, no. 1: 94–101. 10.1007/s12020-019-01882-4. 30840228

[fsn371473-bib-0008] Boef, A. G. , O. M. Dekkers , and S. le Cessie . 2015. “Mendelian Randomization Studies: A Review of the Approaches Used and the Quality of Reporting.” International Journal of Epidemiology 44, no. 2: 496–511. 10.1093/ije/dyv071. 25953784

[fsn371473-bib-0009] Bosetti, C. , L. Kolonel , E. Negri , et al. 2001. “A Pooled Analysis of Case‐Control Studies of Thyroid Cancer. VI. Fish and Shellfish Consumption.” Cancer Causes & Control 12, no. 4: 375–382. 10.1023/a:1011267123398. 11456234

[fsn371473-bib-0010] Bounds, W. , K. W. Betzing , R. M. Stewart , and R. F. Holcombe . 1994. “Social Drinking and the Immune Response: Impairment of Lymphokine‐Activated Killer Activity.” American Journal of the Medical Sciences 307, no. 6: 391–395. 10.1097/00000441-199406000-00001. 8198143

[fsn371473-bib-0011] Bowden, J. , G. Davey Smith , P. C. Haycock , and S. Burgess . 2016. “Consistent Estimation in Mendelian Randomization With Some Invalid Instruments Using a Weighted Median Estimator.” Genetic Epidemiology 40, no. 4: 304–314. 10.1002/gepi.21965. 27061298 PMC4849733

[fsn371473-bib-0012] Bulik‐Sullivan, B. , H. K. Finucane , V. Anttila , et al. 2015. “An Atlas of Genetic Correlations Across Human Diseases and Traits.” Nature Genetics 47, no. 11: 1236–1241. 10.1038/ng.3406. 26414676 PMC4797329

[fsn371473-bib-0013] Bulik‐Sullivan, B. K. , P. R. Loh , H. K. Finucane , et al. 2015. “LD Score Regression Distinguishes Confounding From Polygenicity in Genome‐Wide Association Studies.” Nature Genetics 47, no. 3: 291–295. 10.1038/ng.3211. 25642630 PMC4495769

[fsn371473-bib-0014] Burgess, S. , A. Butterworth , and S. G. Thompson . 2013. “Mendelian Randomization Analysis With Multiple Genetic Variants Using Summarized Data.” Genetic Epidemiology 37, no. 7: 658–665. 10.1002/gepi.21758. 24114802 PMC4377079

[fsn371473-bib-0015] Burgess, S. , R. A. Scott , N. J. Timpson , G. Davey Smith , and S. G. Thompson . 2015. “Using Published Data in Mendelian Randomization: A Blueprint for Efficient Identification of Causal Risk Factors.” European Journal of Epidemiology 30, no. 7: 543–552. 10.1007/s10654-015-0011-z. 25773750 PMC4516908

[fsn371473-bib-0016] Burgess, S. , and S. G. Thompson . 2017. “Interpreting Findings From Mendelian Randomization Using the MR‐Egger Method.” European Journal of Epidemiology 32, no. 5: 377–389. 10.1007/s10654-017-0255-x. 28527048 PMC5506233

[fsn371473-bib-0017] Burgess, S. , and S. G. Thompson . 2015. “Multivariable Mendelian Randomization: The Use of Pleiotropic Genetic Variants to Estimate Causal Effects.” American Journal of Epidemiology 181, no. 4: 251–260. 10.1093/aje/kwu283. 25632051 PMC4325677

[fsn371473-bib-0018] Bycroft, C. , C. Freeman , D. Petkova , et al. 2018. “The UK Biobank Resource With Deep Phenotyping and Genomic Data.” Nature 562, no. 7726: 203–209. 10.1038/s41586-018-0579-z. 30305743 PMC6786975

[fsn371473-bib-0019] Calder, P. C. 2017. “Omega‐3 Fatty Acids and Inflammatory Processes: From Molecules to Man.” Biochemical Society Transactions 45, no. 5: 1105–1115. 10.1042/bst20160474. 28900017

[fsn371473-bib-0020] Carlé, A. , I. Bülow Pedersen , N. Knudsen , et al. 2013. “Graves' Hyperthyroidism and Moderate Alcohol Consumption: Evidence for Disease Prevention.” Clinical Endocrinology 79, no. 1: 111–119. 10.1111/cen.12106. 23170908

[fsn371473-bib-0021] Carlé, A. , I. B. Pedersen , N. Knudsen , et al. 2012. “Moderate Alcohol Consumption May Protect Against Overt Autoimmune Hypothyroidism: A Population‐Based Case‐Control Study.” European Journal of Endocrinology 167, no. 4: 483–490. 10.1530/eje-12-0356. 22802427

[fsn371473-bib-0022] Caslin, B. , K. Mohler , S. Thiagarajan , and E. Melamed . 2021. “Alcohol as Friend or Foe in Autoimmune Diseases: A Role for Gut Microbiome?” Gut Microbes 13, no. 1: 1916278. 10.1080/19490976.2021.1916278. 34224314 PMC8259720

[fsn371473-bib-0023] Chaker, L. , A. C. Bianco , J. Jonklaas , and R. P. Peeters . 2017. “Hypothyroidism.” Lancet 390, no. 10101: 1550–1562. 10.1016/s0140-6736(17)30703-1. 28336049 PMC6619426

[fsn371473-bib-0024] Charpentier, B. , D. Franco , L. Paci , et al. 1984. “Deficient Natural Killer Cell Activity in Alcoholic Cirrhosis.” Clinical and Experimental Immunology 58, no. 1: 107–115. 6236915 PMC1576962

[fsn371473-bib-0025] Chen, D. W. , B. H. H. Lang , D. S. A. McLeod , K. Newbold , and M. R. Haymart . 2023. “Thyroid Cancer.” Lancet 401, no. 10387: 1531–1544. 10.1016/s0140-6736(23)00020-x. 37023783

[fsn371473-bib-0026] Choi, W. J. , and J. Kim . 2014. “Dietary Factors and the Risk of Thyroid Cancer: A Review.” Clinical Nutrition Research 3, no. 2: 75–88. 10.7762/cnr.2014.3.2.75. 25136535 PMC4135245

[fsn371473-bib-0027] Davey Smith, G. , and G. Hemani . 2014. “Mendelian Randomization: Genetic Anchors for Causal Inference in Epidemiological Studies.” Human Molecular Genetics 23, no. R1: R89–R98. 10.1093/hmg/ddu328. 25064373 PMC4170722

[fsn371473-bib-0028] Davies, T. F. , S. Andersen , R. Latif , et al. 2020. “Graves' Disease.” Nature Reviews. Disease Primers 6, no. 1: 52. 10.1038/s41572-020-0184-y. 32616746

[fsn371473-bib-0029] De Leo, S. , S. Y. Lee , and L. E. Braverman . 2016. “Hyperthyroidism.” Lancet 388, no. 10047: 906–918. 10.1016/s0140-6736(16)00278-6. 27038492 PMC5014602

[fsn371473-bib-0030] Du, Q. , and W. Shen . 2023. “Research Progress of Plant‐Derived Natural Products in Thyroid Carcinoma.” Frontiers in Chemistry 11: 1279384. 10.3389/fchem.2023.1279384. 38268761 PMC10806030

[fsn371473-bib-0031] Echeverry‐Alzate, V. , K. M. Bühler , J. Calleja‐Conde , et al. 2019. “Adult‐Onset Hypothyroidism Increases Ethanol Consumption.” Psychopharmacology 236, no. 4: 1187–1197. 10.1007/s00213-018-5123-1. 30470859

[fsn371473-bib-0032] Effraimidis, G. , J. G. Tijssen , and W. M. Wiersinga . 2012. “Alcohol Consumption as a Risk Factor for Autoimmune Thyroid Disease: A Prospective Study.” European Thyroid Journal 1, no. 2: 99–104. 10.1159/000338920. 24783003 PMC3821464

[fsn371473-bib-0033] Esfahani, K. S. , N. Asri , M. Mahmoudi Ghehsareh , M. Rezaei‐Tavirani , S. Jahani‐Sherafat , and M. Rostami‐Nejad . 2024. “The Role of Gluten in the Development of Autoimmune Thyroid Diseases: A Narrative Review.” International Journal of Endocrinology and Metabolism 22, no. 3: e153730. 10.5812/ijem-153730. 40065831 PMC11892518

[fsn371473-bib-0034] Grani, G. , M. Sponziello , S. Filetti , and C. Durante . 2024. “Thyroid Nodules: Diagnosis and Management.” Nature Reviews. Endocrinology 20, no. 12: 715–728. 10.1038/s41574-024-01025-4. 39152228

[fsn371473-bib-0035] Hartwig, F. P. , G. Davey Smith , and J. Bowden . 2017. “Robust Inference in Summary Data Mendelian Randomization via the Zero Modal Pleiotropy Assumption.” International Journal of Epidemiology 46, no. 6: 1985–1998. 10.1093/ije/dyx102. 29040600 PMC5837715

[fsn371473-bib-0036] Hemani, G. , J. Zheng , B. Elsworth , et al. 2018. “The MR‐Base Platform Supports Systematic Causal Inference Across the Human Phenome.” eLife 7: 34408. 10.7554/eLife.34408. PMC5976434 29846171

[fsn371473-bib-0037] Hess, J. M. , C. B. Stephensen , M. Kratz , and B. W. Bolling . 2021. “Exploring the Links Between Diet and Inflammation: Dairy Foods as Case Studies.” Advances in Nutrition 12, no. Suppl 1: 1s–13s. 10.1093/advances/nmab108. 34632478 PMC8502778

[fsn371473-bib-0038] Howard, D. M. , M. J. Adams , T. K. Clarke , et al. 2019. “Genome‐Wide Meta‐Analysis of Depression Identifies 102 Independent Variants and Highlights the Importance of the Prefrontal Brain Regions.” Nature Neuroscience 22, no. 3: 343–352. 10.1038/s41593-018-0326-7. 30718901 PMC6522363

[fsn371473-bib-0039] Howe, L. J. , M. G. Nivard , T. T. Morris , et al. 2022. “Within‐Sibship Genome‐Wide Association Analyses Decrease Bias in Estimates of Direct Genetic Effects.” Nature Genetics 54, no. 5: 581–592. 10.1038/s41588-022-01062-7. 35534559 PMC9110300

[fsn371473-bib-0040] Huang, J. , C. H. Ngai , Y. Deng , et al. 2023. “Incidence and Mortality of Thyroid Cancer in 50 Countries: A Joinpoint Regression Analysis of Global Trends.” Endocrine 80, no. 2: 355–365. 10.1007/s12020-022-03274-7. 36607509

[fsn371473-bib-0041] Jiang, L. , Z. Zheng , H. Fang , and J. Yang . 2021. “A Generalized Linear Mixed Model Association Tool for Biobank‐Scale Data.” Nature Genetics 53, no. 11: 1616–1621. 10.1038/s41588-021-00954-4. 34737426

[fsn371473-bib-0042] Jung, S. K. , K. Kim , K. Tae , G. Kong , and M. K. Kim . 2013. “The Effect of Raw Vegetable and Fruit Intake on Thyroid Cancer Risk Among Women: A Case‐Control Study in South Korea.” British Journal of Nutrition 109, no. 1: 118–128. 10.1017/s0007114512000591. 22455656

[fsn371473-bib-0043] Kalra, S. , S. Aggarwal , and D. Khandelwal . 2019. “Thyroid Dysfunction and Type 2 Diabetes Mellitus: Screening Strategies and Implications for Management.” Diabetes Therapy 10, no. 6: 2035–2044. 10.1007/s13300-019-00700-4. 31583645 PMC6848627

[fsn371473-bib-0044] Kim, D. S. , and S. Park . 2023. “Interactions Between Polygenetic Variants and Lifestyle Factors in Hypothyroidism: A Hospital‐Based Cohort Study.” Nutrients 15, no. 17: 3850. 10.3390/nu15173850. 37686882 PMC10490100

[fsn371473-bib-0045] Kurki, M. I. , J. Karjalainen , P. Palta , et al. 2023. “FinnGen Provides Genetic Insights From a Well‐Phenotyped Isolated Population.” Nature 613, no. 7944: 508–518. 10.1038/s41586-022-05473-8. 36653562 PMC9849126

[fsn371473-bib-0046] Lancet . 2012. “Thyroid Disease‐More Research Needed.” Lancet 379, no. 9821: 1076. 10.1016/s0140-6736(12)60445-0. 22444390

[fsn371473-bib-0047] Li, X. , Z. Chen , L. Wu , P. Tu , Z. Mo , and M. Xing . 2024. “Prevalence of Thyroid Nodule and Relationship With Physiological and Psychosocial Factors Among Adults in Zhejiang Province, China: A Baseline Survey of a Cohort Study.” BMC Public Health 24, no. 1: 1854. 10.1186/s12889-024-19375-z. 38992649 PMC11238450

[fsn371473-bib-0048] Loya, H. , G. Kalantzis , F. Cooper , and P. F. Palamara . 2025. “A Scalable Variational Inference Approach for Increased Mixed‐Model Association Power.” Nature Genetics 57, no. 2: 461–468. 10.1038/s41588-024-02044-7. 39789286 PMC11821521

[fsn371473-bib-0049] Lu, M. D. , H. Li , J. H. Nie , et al. 2022. “Dual Inhibition of BRAF‐MAPK and STAT3 Signaling Pathways in Resveratrol‐Suppressed Anaplastic Thyroid Cancer Cells With BRAF Mutations.” International Journal of Molecular Sciences 23, no. 22: 14385. 10.3390/ijms232214385. 36430869 PMC9692422

[fsn371473-bib-0050] Memon, A. , A. Varghese , and A. Suresh . 2002. “Benign Thyroid Disease and Dietary Factors in Thyroid Cancer: A Case‐Control Study in Kuwait.” British Journal of Cancer 86, no. 11: 1745–1750. 10.1038/sj.bjc.6600303. 12087461 PMC2375394

[fsn371473-bib-0051] Nigra, A. E. , K. E. Nachman , D. C. Love , M. Grau‐Perez , and A. Navas‐Acien . 2017. “Poultry Consumption and Arsenic Exposure in the U.S. Population.” Environmental Health Perspectives 125, no. 3: 370–377. 10.1289/ehp351. 27735790 PMC5332189

[fsn371473-bib-0052] Niwattisaiwong, S. , K. D. Burman , and M. Li‐Ng . 2017. “Iodine Deficiency: Clinical Implications.” Cleveland Clinic Journal of Medicine 84, no. 3: 236–244. 10.3949/ccjm.84a.15053. 28322679

[fsn371473-bib-0053] Özgüç Çömlek, F. , and M. K. Körez . 2025. “Can Consumption of Traditional Fermented Foods Protect Against Hashimoto's Thyroiditis?” Nutrición Hospitalaria 43: 428–436. 10.20960/nh.05508. 40195756

[fsn371473-bib-0054] Palmer, T. M. , D. A. Lawlor , R. M. Harbord , et al. 2012. “Using Multiple Genetic Variants as Instrumental Variables for Modifiable Risk Factors.” Statistical Methods in Medical Research 21, no. 3: 223–242. 10.1177/0962280210394459. 21216802 PMC3917707

[fsn371473-bib-0055] Pearce, E. N. , A. P. Farwell , and L. E. Braverman . 2003. “Thyroiditis.” New England Journal of Medicine 348, no. 26: 2646–2655. 10.1056/NEJMra021194. 12826640

[fsn371473-bib-0056] Pierce, B. L. , H. Ahsan , and T. J. Vanderweele . 2011. “Power and Instrument Strength Requirements for Mendelian Randomization Studies Using Multiple Genetic Variants.” International Journal of Epidemiology 40, no. 3: 740–752. 10.1093/ije/dyq151. 20813862 PMC3147064

[fsn371473-bib-0057] Rangel, A. , D. Bezerra , D. C. Sales , et al. 2023. “An Overview of the Occurrence of Bioactive Peptides in Different Types of Cheeses.” Food 12, no. 23: 4261. 10.3390/foods12234261. PMC10706718 38231707

[fsn371473-bib-0058] Refaat, B. , and F. Azzeh . 2021. “Factors Associated With Thyroid Disorders and Iodine Adequacy in Pregnant Saudi Women.” Biological Trace Element Research 199, no. 5: 1715–1728. 10.1007/s12011-020-02301-w. 32710351

[fsn371473-bib-0059] Ruggeri, R. M. , M. C. Barbalace , L. Croce , et al. 2023. “Autoimmune Thyroid Disorders: The Mediterranean Diet as a Protective Choice.” Nutrients 15, no. 18: 3953. 10.3390/nu15183953. 37764737 PMC10535745

[fsn371473-bib-0060] Ruggeri, R. M. , S. Giovinazzo , M. C. Barbalace , et al. 2021. “Influence of Dietary Habits on Oxidative Stress Markers in Hashimoto's Thyroiditis.” Thyroid 31, no. 1: 96–105. 10.1089/thy.2020.0299. 32729374

[fsn371473-bib-0061] Sanderson, E. , G. Davey Smith , F. Windmeijer , and J. Bowden . 2019. “An Examination of Multivariable Mendelian Randomization in the Single‐Sample and Two‐Sample Summary Data Settings.” International Journal of Epidemiology 48, no. 3: 713–727. 10.1093/ije/dyy262. 30535378 PMC6734942

[fsn371473-bib-0062] Tang, Y. , T. Yan , G. Wang , et al. 2017. “Correlation Between Insulin Resistance and Thyroid Nodule in Type 2 Diabetes Mellitus.” International Journal of Endocrinology 2017: 1617458. 10.1155/2017/1617458. 29158735 PMC5660821

[fsn371473-bib-0063] Taylor, P. N. , D. Albrecht , A. Scholz , et al. 2018. “Global Epidemiology of Hyperthyroidism and Hypothyroidism.” Nature Reviews. Endocrinology 14, no. 5: 301–316. 10.1038/nrendo.2018.18. 29569622

[fsn371473-bib-0064] Turesky, R. J. 2018. “Mechanistic Evidence for Red Meat and Processed Meat Intake and Cancer Risk: A Follow‐Up on the International Agency for Research on Cancer Evaluation of 2015.” Chimia (Aarau) 72, no. 10: 718–724. 10.2533/chimia.2018.718. 30376922 PMC6294997

[fsn371473-bib-0065] Unal Kocabas, G. , A. Kisim Blatti , A. Berdeli , A. G. Ozgen , and B. Sarer Yurekli . 2024. “MAPK Pathway and NIS in B‐CPAP Human Papillary Thyroid Carcinoma Cells Treated With Resveratrol.” Pathology, Research and Practice 263: 155623. 10.1016/j.prp.2024.155623. 39405802

[fsn371473-bib-0066] Uppal, N. , R. Collins , and B. James . 2023. “Thyroid Nodules: Global, Economic, and Personal Burdens.” Frontiers in Endocrinology (Lausanne) 14: 1113977. 10.3389/fendo.2023.1113977. PMC9899850 36755911

[fsn371473-bib-0067] Verbanck, M. , C. Y. Chen , B. Neale , and R. Do . 2018. “Publisher Correction: Detection of Widespread Horizontal Pleiotropy in Causal Relationships Inferred From Mendelian Randomization Between Complex Traits and Diseases.” Nature Genetics 50, no. 8: 1196. 10.1038/s41588-018-0164-2. 29967445

[fsn371473-bib-0068] Voulgari, P. V. , A. I. Venetsanopoulou , N. Kalpourtzi , et al. 2022. “Thyroid Dysfunction in Greece: Results From the National Health Examination Survey EMENO.” PLoS One 17, no. 3: e0264388. 10.1371/journal.pone.0264388. 35245310 PMC8896672

[fsn371473-bib-0069] Walczak, K. , and L. Sieminska . 2021. “Obesity and Thyroid Axis.” International Journal of Environmental Research and Public Health 18, no. 18: 9434. 10.3390/ijerph18189434. 34574358 PMC8467528

[fsn371473-bib-0070] Wan, Z. , Y. Li , X. Dong , et al. 2023. “Influence of Metabolic Syndrome and Lifestyle Factors on Thyroid Nodules in Chinese Adult Men: A Cross‐Sectional Study.” European Thyroid Journal 12, no. 6: 168. 10.1530/etj-23-0168. PMC10692680 37728058

[fsn371473-bib-0071] Wang, Y. , J. Wang , Z. Chen , et al. 2021. “Analysis of the Correlation Between High Iodized Salt Intake and the Risk of Thyroid Nodules: A Large Retrospective Study.” BMC Cancer 21, no. 1: 1000. 10.1186/s12885-021-08700-z. 34493230 PMC8425165

[fsn371473-bib-0072] Wiersinga, W. M. 2016. “Clinical Relevance of Environmental Factors in the Pathogenesis of Autoimmune Thyroid Disease.” Endocrinology and Metabolism (Seoul) 31, no. 2: 213–222. 10.3803/EnM.2016.31.2.213. PMC4923404 27184015

[fsn371473-bib-0073] Zamora, E. A. , S. Khare , and S. Cassaro . 2025. “Thyroid Nodule.” In StatPearls. StatPearls Publishing Copyright 2025, StatPearls Publishing LLC. 30571043

[fsn371473-bib-0074] Zamwar, U. M. , and K. N. Muneshwar . 2023. “Epidemiology, Types, Causes, Clinical Presentation, Diagnosis, and Treatment of Hypothyroidism.” Cureus 15, no. 9: e46241. 10.7759/cureus.46241. 37908940 PMC10613832

[fsn371473-bib-0075] Zhang, M. , X. Dong , Z. Huang , et al. 2023. “Cheese Consumption and Multiple Health Outcomes: An Umbrella Review and Updated Meta‐Analysis of Prospective Studies.” Advances in Nutrition 14, no. 5: 1170–1186. 10.1016/j.advnut.2023.06.007. 37328108 PMC10509445

[fsn371473-bib-0076] Zhang, X. , Q. Lu , Y. Luo , L. Wang , Y. Tian , and X. Luo . 2024. “The Causal Relationship Between Major Depression Disorder and Thyroid Diseases: A Mendelian Randomization Study and Mediation Analysis.” Journal of Affective Disorders 359: 287–299. 10.1016/j.jad.2024.05.097. 38788859

[fsn371473-bib-0077] Zhang, X. , X. Wang , H. Hu , H. Qu , Y. Xu , and Q. Li . 2023. “Prevalence and Trends of Thyroid Disease Among Adults, 1999–2018.” Endocrine Practice 29, no. 11: 875–880. 10.1016/j.eprac.2023.08.006. 37619827

[fsn371473-bib-0078] Żółkiewicz, J. , A. Marzec , M. Ruszczyński , and W. Feleszko . 2020. “Postbiotics‐A Step Beyond Pre‐ and Probiotics.” Nutrients 12, no. 8: 2189. 10.3390/nu12082189. 32717965 PMC7468815

